# Acetyl-bufalin shows potent efficacy against non-small-cell lung cancer by targeting the CDK9/STAT3 signalling pathway

**DOI:** 10.1038/s41416-020-01135-6

**Published:** 2020-10-30

**Authors:** Lehe Yang, Feng Zhou, Yan Zhuang, Yanan Liu, Lingyuan Xu, Haiyang Zhao, Youqun Xiang, Xuanxuan Dai, Zhiguo Liu, Xiaoying Huang, Liangxing Wang, Chengguang Zhao

**Affiliations:** 1 School of Pharmaceutical Sciences, Wenzhou Medical University, 325035 Wenzhou, Zhejiang China; 2 The First Affiliated Hospital, Wenzhou Medical University, 325000 Wenzhou, Zhejiang China; 3 The Institute of Life Sciences, Wenzhou University, 325035 Wenzhou, Zhejiang China

**Keywords:** Cancer, Drug development

## Abstract

**Background:**

Cyclin-dependent kinase 9 (CDK9) is a promising prognostic marker and therapeutic target in cancers. Bufalin is an effective anti-tumour agent; however, the clinical application of bufalin is limited due to its high toxicity. Acetyl-bufalin, the bufalin prodrug, was designed and synthesised with higher efficiency and lower toxicity.

**Methods:**

Three non-small-cell lung cancer (NSCLC) cell lines, a xenograft model and a patient-derived xenograft (PDX) model were used to examine the effects of acetyl-bufalin. CDK9/STAT3 involvement was investigated by knockdown with siRNA, proteome microarray assay, western blot analysis and co-immunoprecipitation experiments. Acute toxicity test and pharmacokinetics (PK) study were conducted to assess the safety and PK. The human NSCLC tissues were analysed to verify high CDK9 expression.

**Results:**

We showed that CDK9 induced NSCLC cell proliferation and that this effect was associated with STAT3 activation, specifically an increase in STAT3 phosphorylation and transcription factor activity. Acetyl-bufalin is an effective and safety inhibitor of the CDK9/STAT3 pathway, leading to the impediment of various oncogenic processes in NSCLC. Molecular docking and high-throughput proteomics platform analysis uncovered acetyl-bufalin directly binds to CDK9. Consequently, acetyl-bufalin impaired the complex formation of CDK9 and STAT3, decreased the expressions of P-STAT3, and transcribed target genes such as cyclin B1, CDC2, MCL-1, Survivin, VEGF, BCL2, and it upregulated the expression levels of BAX and caspase-3 activity. Acetyl-bufalin inhibited tumour growth in NSCLC xenograft and PDX models.

**Conclusions:**

Acetyl-bufalin is a novel blocker of the CDK9/STAT3 pathway thus may have potential in therapy of NSCLC and other cancers.

## Background

Chansu, the dried skin secretions of the giant toads, *Bufo bufo gargarizans* Cantor and *Bufo melanostictus* Schneider, is an important traditional Chinese medicine that is widely used in China and other Asian countries to clinically treat several ailments.^1^ Cinobufacini (Huachansu) is a water-soluble extract from the skins and parotid venom glands of the toad, *Bufo bufo gargarizans* Cantor, which has been approved by the Chinese State Food and Drug Administration (SFDA; IS09002).^2,3^ Cinobufacini is widely used to treat patients with liver, lung, colon and pancreatic cancers at oncology clinics in China.^4–6^ However, the pharmacological constituents and precise mechanisms of cinobufacini require further study, and its active compounds remain unclear.^7^ We screened the effective anti-tumour properties of 13 ingredients from toad skin on non-small-cell lung cancer (NSCLC) cells and found that bufalin was the most effective compound against NSCLC cell proliferation (Supplementary Table 1).

Bufalin is the prominent active constituent of chansu and cinobufacini.^8,9^ Pharmacological studies have shown that bufalin possesses significant anti-tumour effects.^10,11^ Although bufalin display perfectly capable of killing various tumour cells in vitro, however, it is always obtained unsatisfactory results after administration in vivo, indicating the obvious flaw in the drugability of bufalin.^12,13^ Because of its fast metabolism, toxicity, insolubility in water and short half-life, its application in clinics is limited.^14^ However, studies have shown that the substituent group could influence the bufalin metabolic enzyme interactions; thereafter, the kinetic behaviour and parameters were varied greatly.^15^ In this study, the bufalin prodrug, acetyl-bufalin, was designed and synthesised base on aspirin (acetylsalicylic acid) and diacerein (diacetoxyrhein) to develop new analogues with higher efficiency and lower toxicity (Supplementary Fig. S1). Acetyl-bufalin can help to optimise drug delivery and is expected to improve pharmacokinetics and anti-tumour activity.

Cyclin-dependent kinase 9 (CDK9) is a key transcriptional regulator and promising prognostic marker and therapeutic target in cancers.^16,17^ Unlike other CDKs, CDK9 does not regulate the cell cycle but promotes RNA synthesis in the genetic processes of cell growth, differentiation and viral pathogenesis.^18–20^ Previous data indicated that the IL6-inducible complex of CDK9/STAT3 is essential for inducing γ-fibrinogen during the hepatic acute-phase response.^21^ Therefore, using CDK9 inhibitors to disrupt IL6/CDK9/STAT3 signalling could be a potential therapeutic strategy for treating inflammation and cancer. In this study, IL6 strongly induced the CDK9 and STAT3 complex in NSCLC cells. Acetyl-bufalin abolished IL6-inducible P-STAT3. Moreover, acetyl-bufalin inhibited CDK9 expression and decreased phospho-RNA Pol II Ser2 in vitro and in vivo. Here, we demonstrate that acetyl-bufalin is a novel inhibitor of the CDK9/STAT3 axis.

## Methods

### Patient tissues

With the approval and informed consent of the Ethics Committee of the First Affiliated Hospital of Wenzhou Medical University, a total of 12 pair human NSCLC tissues and their adjacent tissues were collected from the First Affiliated Hospital of Wenzhou Medical University. Fresh tissues were immediately snap-frozen and stored at −80 °C or fixed and embedded in paraffin (Supplementary Table 2).

### Cell culture

Human NSCLC cell lines PC-9, H460 and A549 were purchased from the Shanghai Institute of Biosciences and Cell Resources Center (Chinese Academy of Sciences, Shanghai, China). All cells were cultured in Roswell Park Memorial Institute (RPMI)-1640 medium (Thermo Fisher Scientific) with 10% foetal bovine serum (FBS; Thermo Fisher Scientific). The cells were cultured in a humidified cell incubator at 37 °C with 5% CO_2_.

### Antibodies and reagents

The antibodies against P-CDK9, CDK9, P-STAT3, BAX, S2 RNAPII and RNAPII were purchased from Abcam Co. (Cambridge, UK); The antibodies against STAT3, MCL-1, VEGF, Survivin, Cyclin B1, CDC2 and GAPDH were purchased from Cell Signal Technology (Danvers, MA, USA). The antibodies against BCL2, horseradish peroxidase (HRP)-conjugated donkey anti-rabbit IgG and HRP-conjugated goat anti-mouse IgG were purchased from Santa Cruz Biotechnology Inc. (Dallas, TX, USA). Dimethyl sulfoxide (DMSO) and methylthiazolyldiphenyl-tetrazolium bromide (MTT) were purchased from Sigma-Aldrich Co. (St Louis, MO, USA). A protease phosphatase-inhibitor mixture was obtained from Applygen Technologies (Beijing, China). The caspase‐3 colorimetric assay kit was purchased from Abcam Co. (Cambridge, UK). The Annexin V-FITC apoptosis Detection Kit I and propidium iodide (PI) were purchased from BD Pharmingen (Franklin Lakes, NJ, USA). A Bradford protein-assay kit, enhanced chemiluminescence kit and polyvinylidene fluoride (PVDF) membrane were obtained from Bio-Rad Laboratories (Hercules, CA, USA). Coomassie Brilliant Blue, acrylamide (30%), tetramethylethylenediamine, Tris-glycine, sodium dodecyl sulfate, pre-stained protein marker and non-fat dry milk were from obtained Bio-Rad Laboratories. Bufalin was purchased from Herbest Biological Technology Co. (Baoji, China). SNS-032 was purchased from Selleck Chemicals (Houston, USA). The compounds used in vitro were dissolved in DMSO.

### MTT cytotoxicity assay

The cytotoxicity and viability of human NSCLC cells and normal cells were determined by MTT assay. MTT assay was performed as described previously.^22^ MTT assay was used to determine the cell cytotoxicity and viability in human NSCLC cells. Cells (5 × 10^3^ cells/well) were plated in 96-well plates and attached overnight. After the proper treatment, the MTT solution was added at 25 µL/well and incubated at 37 °C for 4 h. The formazan crystals were dissolved in 150 µL of DMSO, and the optical density (OD) was measured using a Microplate Reader at 490 nm. The cell viability was calculated by the following formula: viability = (average OD values of treatment wells/average OD values of vehicle control wells) × 100%. And half-maximal inhibitory concentration (IC_50_) values were determined by GraphPad Pro Prism 7.0.

### Colony-formation assay

The human NSCLC cells and normal cells were seeded at 1000 cells/well on six-well plates, placed in 5% CO_2_ atmosphere and cultured overnight at 37 °C. DMSO (Control) and acetyl-bufalin (50, 100, 200 nM) were added to the cells for 24 h. The fresh medium was replaced every 2 days to maintain the growth of cells for 7 days. Colonies were washed with phosphate-buffered saline (PBS), fixed with 4% paraformaldehyde at room temperature for 15 min, washed with purified water for three times and stained with Crystal violet for 10 min. Each experiment was in triplicate, and three independent experiments were carried out.

### Wound-healing migration assay

The ability of cell migration was evaluated by wound-healing assay. The cells grew to 80–90% confluence in six-well plates and then scraped the cell monolayer at the centre of the wells with the tip of a sterile 10 μL pipette to form a clean, straight wound area. Then, the wells were washed with PBS to remove detached cells from the plates. After that, cells were cultured in serum-free RPMI medium with vehicle control or acetyl-bufalin (25, 50, 100 nM). The migration of cells to the wound area was photographed at the 0, 24 and 48 h timepoints with a microscope and imaging system (Leica, Wetzlar, Germany).

### Acute toxicity test

In the acute toxicity test, 30 NIH mice (female, 8 weeks old) were randomly divided into three groups (*n* = 10), including vehicle group, bufalin group (30 mg/kg) and acetyl-bufalin group (30 mg/kg). The three groups received intraperitoneal injection only on the first day. All the mice were housed under 12 h light–dark cycles at 25 °C and free for water and diet. In addition, the mortality and weight of the mice were observed for 14 days. All the mice were sacrificed by cervical dislocation.

### Pharmacokinetic study

For the pharmacokinetic study, female S.D. rats (body weight 200 ± 10 g) have fasted overnight with free access to water before the experiment. Twelve rats were randomly divided into four groups. The time schedule included eight timepoints, and three S.D. rats were sampled at each time-point. Bufalin and acetyl-bufalin were administered intravenously and intraperitoneally to the rats at the dose of 0.25 mg/kg and 2 mg/kg, respectively. The animals had free access to water during the experiment. Blood samples were collected from the retro-orbital plexus of each rat at 0.083, 0.25, 0.5, 1, 2, 4, 6, 8, 12, 24 h after administration. Plasma was separated from the blood after centrifugation at 6000×*g* for 10 min and frozen at −20 °C until analysis. In total, 20 µL of standard samples in duplicate, quality control samples in duplicate and rat plasma samples were mixed with 60 µL of ethyl acetate containing IS (200 ng/mL of tolbutamide, 50 ng/mL of propranolol and 500 ng/mL of Dic) in EP tubes. After the mixture was vortexed for 1 min, then centrifuged for 10 min (13,000 rpm, 4 °C), transferred 50 µL supernatant to a 96-well plate with 150 µL purred water, shacked for 10 min and finally injected 5 µL into liquid chromatographic-tandem mass spectrometry (LC-MS/MS) system for analysis.

### Cell apoptosis assay

Cell apoptosis assay was performed as described previously.^23^ The human NSCLC cells and HUVEC cells were inoculated in six-well plates, cultured in complete medium to grow to 80% confluency, then treated with DMSO (Control) and acetyl-bufalin (50, 100, 200 nM) for 24 h to evaluate the effects of acetyl-bufalin on cell apoptosis. Cells were collected, washed twice in ice-cold PBS and then resuspended in the binding buffer according to the instructions of the apoptosis Kit. The treated cells (as described above) were simultaneously incubated with fluorescein-labelled Annexin V and PI. Annexin V-binding buffer was then added to the mixture, and fluorescence was measured on a FACSCalibur (BD Biosciences; Baltimore, MD, USA). Flowjo software was used to analyse the data.

### Cell cycle assay

The PC-9 cells were seeded into six-well plates for 12 h, and then treated with DMSO (Control) or acetyl-bufalin (50, 100, 200 nM) for 24 h. Cells were then labelled with PI, and the cell cycle was analysed on a FACSCalibur. Flowjo software was used to analyse the data.

### Hoechst 33342 staining

A Hoechst 33342 assay kit (Beyotime Institute of Biotechnology, China) was used to detect apoptosis in H460 and PC-9 cells. Cells were inoculated in six-well plates and incubated with DMSO (Control) and acetyl-bufalin (50, 100, 200 nM). After 24 h, cells were washed in PBS and fixed in freshly prepared 4% paraformaldehyde for 15 min. Then the cells were washed with PBS again, incubated with Hoechst 33342 staining solution for 20 min, and washed with PBS before anti-fade mounting medium was added. Apoptotic cells were detected using a fluorescence microscope (Leica, Wetzlar, Germany).

### Western blot analysis

Tissue proteins were extracted with tissue protein lysate buffer. Cells were grown to different densities and extracted with cell protein lysate buffer. The protein was mixed with loading buffer and boiled for 10 min at 100 °C, then loaded and separated by 10% or 12% sodium dodecyl sulfate-polyacrylamide gel (SDS-PAGE) and then transferred onto a PVDF membrane blocking with 5% skim milk. The blots were incubated overnight with specific primary antibodies at 4 °C. On the second day, after washing with TBST (a mixture of tris-buffered saline (TBS) and Tween 20) three times, the nitrocellulose membranes were incubated with horseradish peroxidase-conjugated secondary antibodies for 1 h. The immunoreactive bands were visualised with enhanced chemiluminescence reagent.

### Caspase-3 activity assay

The caspase-3 assay was performed as described previously.^23^ According to the manufacturer’s protocol, the activity of caspase‐3 was evaluated by using a caspase‐3 colorimetric assay kit (Abcam, Cambridge, UK). H460 and PC-9 cells were collected and resuspended in lysis buffer. After incubation on ice for 10 min, the cell lysate was centrifuged at 12,000 rpm for 10 min at 4 °C, and the protein concentration in the supernatants was measured using the Bradford dye method. The supernatants were incubated with a reaction buffer containing 200 µM devd-p-nitroanilide, for caspase‐3 in a caspase assay buffer at 37 °C with 10 mM dithiothreitol for 2 h. The activity of caspase-3 was determined by measuring the absorbance at 405 nm. Each experiment was done in triplicate for three independent experiments.

### STAT3 luciferase-report assay

STAT3 activation was detected by the STAT3 luciferase reporter plasmid (PGLSTAT3-LUC), as was previously described.^23^ Simply put, PC-9 and H460 cells were seeded in 24-well plates and incubated for 24 h before transfection. The cells were grown to different densities and co-transfected with pGLSTAT3-Luc and pRL-TK (a plasmid encoding Renilla luciferase) for 6 h using Lipofectamine 3000 (Invitrogen, Carlsbad, CA, USA). Finally, the cells were treated with different concentrations of gracillin for 12 h. Luciferase activity was assessed by SpectraMax ID3 (Molecular Devices, San Jose, CA, USA). The inhibition of STAT3 activity by acetyl-bufalin was calculated as the ratio between the value of firefly and Renilla luciferase activity. Each experiment was done in triplicate in three independent experiments.

### Proteome microarray assay and data analysis

Arrayit HuProt™ v2.0 19 K Human Proteome Microarrays (CDI Laboratories, Baltimore, MD) were blocked for 1 h with 3% BSA at room temperature. Biotinylated bufalin was diluted to 10 μM in blocking buffer and cultured on the proteome microarray at room temperature for 1 h. The arrays were washed with PBST (a mixture of PBS and Tween 20) and incubated with Cy3-Streptavidin at 1:1000 dilution (Sigma-Aldrich) for 1 h at room temperature. Finally, the microarray was spun dry and scanned with a GenePix 4200 A microarray scanner (Molecular Devices, San Jose, CA). Data were analysed by GenePix Pro 6.0 software. The signal-to-noise ratio (SNR) was defined as the ratio of median foreground value minus median background value. The cut-off value of SNR (>1.1) was set.

### Molecular docking of acetyl-bufalin to the binding spots of CDK9

Molecular docking was performed by using AutoDock Vina 1.0.21.^24,25^ The crystal structure of the human CDK9-A86 complex (PDB code 6GZH) derived from Protein Data Bank was used for our docking study. The input files of ligand and receptor were prepared using Graphical User Interface program AutoDock Tools 1.5.62 (The Scripps Research Institute, CA, USA). During the docking, the receptor was considered as rigid while the ligand was flexible.

### siRNA transfection

The siRNA targeting human CDK9 was purchased from Genepharma (Shanghai, China) with the following sequence: siCDK9-1: 5′-GGCCAAACGUGGACAACUA TT-3′, siCDK9-2: 5′-GAAGGCUGCUAAUGUGCUUTT-3′, siCDK9-3: 5′-CACUGGACCUCAUCGACAA-3′. Cells were transfected with 50 nM siRNA using Lipofectamine 3000 (Invitrogen, CA, USA) for 48 h.

### Co-immunoprecipitation assay

In all, 1 mg of whole-cell extracts were precleared with protein A/G agarose beads (Beyotime Institute of Biotechnology, China) for 2 h at 4 °C. Immunoprecipitation was performed in the presence of the indicated primary antibody at 4 °C overnight. Immune complexes were captured by adding protein A/G agarose beads and rotated at 4 °C for 2 h. After the supernatant was discarded, protein A/G agarose beads were washed with cold PBS for four to five times, and immunoprecipitates were fractionated by SDS-PAGE.

### Animal model

All animal procedures were done accordingly with the Institutional laboratory animal research guidelines and were approved by Wenzhou Medical University Animal Policy and Welfare Committee. Female athymic BALB/c nude mice (5–6 weeks old) and immune-defective non-obese or diabetic severe combined immunodeficiency (NOD-SCID) mice (6–8 weeks old) were purchased from the Vital River Experimental Animal Center (Beijing, China). All the mice were housed under 12 h light–dark cycles at 25 °C and free for water and diet. For the xenograft model, the H460 cells (5 × 10^6^ cells were mixed with an equal volume of PBS and Matrigel in 100 μL) were implanted on the hind flank of nude mice. Once tumour volumes reached ~50 mm^3^, 24 mice were divided into four experimental groups (six mice per group, no differences in mean body weights or tumour volumes between the groups) and intraperitoneal injection with 2 mg/kg SNS-032 (a recognised CDK9 inhibitor), bufalin and acetyl-bufalin every other day. Tumour volume was measured as V = (L × W × W)/2, in which L and W represent the length and width of tumour. Animals were killed by cervical dislocation once the tumour volume reached 1000 mm^3^. The tumours, hearts, livers, kidneys and lungs were removed for use in the histology and western blot analysis.

For the patient-derived human NSCLC xenografts (PDXs) animal model, four pairs of lung cancer samples were obtained from patients who had undergone surgical treatment of lung cancer in the First Affiliated Hospital of Wenzhou Medical University. Fresh tumour tissue was sterilely incised into ~3-mm^3^ pieces and subcutaneously injected into immunodeficient NOD/SCID mice within 30 min. The remaining tumour tissues were stored at −80 °C. When the tumours have successfully engrafted, tumour samples were removed and passaged into the third generation of nude mice for the following studies. All 18 mice were allocated into three groups (*n* = 6) and received i.p. injection of acetyl-bufalin (1 mg/kg/2 days and 2 mg/kg/2 days) as compared with mice injected with PBS (vehicle group) every other day. Tumour volume was measured as V = (L × W × W)/2, in which L and W represent the length and width of tumour. After 14 days, all the mice were killed by cervical dislocation. The tumours, hearts, livers, kidneys and lungs were removed for use in the histology and western blot analysis.

### Immunohistochemistry staining

Tumour tissues were fixed at room temperature in 10% paraformaldehyde and embedded in paraffin. Paraffin-embedded tissue sections were 5 μm thick. Then, the specimens were incubated overnight with anti-CDK9 antibody (Abcam, 1:100 dilution, in 1% bovine serum albumin PBS) at 4 °C. The signal was detected by the corresponding secondary antibody. Thereafter, these slides were stained with diaminobenzidine and counterstained with haematoxylin. The images were captured using a light microscope.

### Haematoxylin and eosin staining

The hearts, lungs, kidneys and livers of animals were fixed in 4% paraformaldehyde and embedded in paraffin. The paraffin tumour tissue sections (5 µm) were deparaffinised and rehydrated and then stained with eosin and haematoxylin. The images were captured using a light microscope.

### Statistical analysis

Data were expressed as mean ± SD of three independent experiments. The statistical differences between different groups were analysed by the Student’s *t* test or one-way analysis of variance in GraphPad Pro7.0 (GraphPad, San Diego, CA). *P* values less than 0.05 (*P* < 0.05) were considered indicative of significance.

## Results

### Acetyl-bufalin changed the pharmacokinetic characteristics of bufalin and enhanced its efficacy

**Fig. 1:**
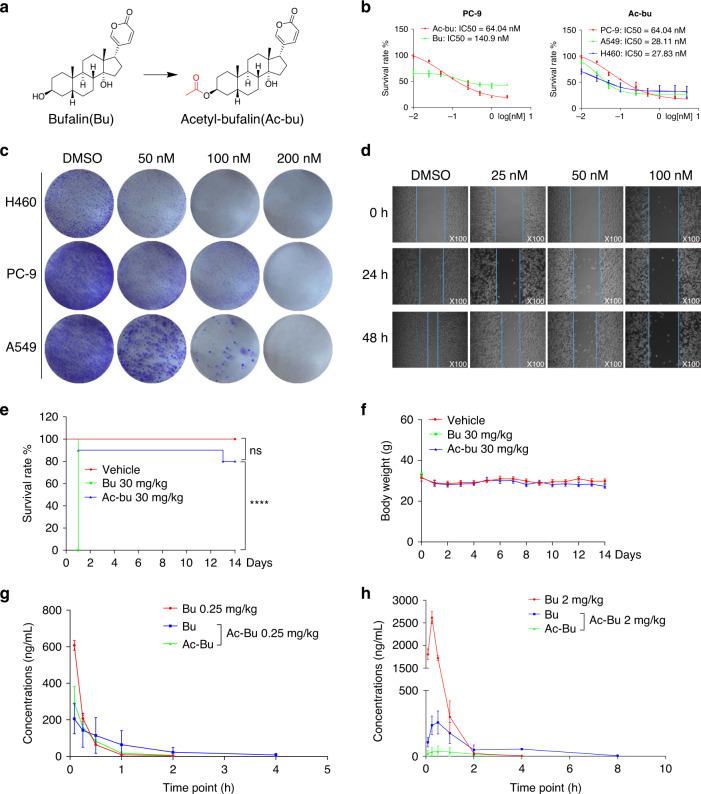
Acetyl-bufalin changed the pharmacokinetic characteristics of bufalin and enhanced its efficacy. **a** Chemical structure of bufalin and acetyl-bufalin. **b** Human non-small-cell lung cancer (NSCLC) cells were incubated with increasing doses of bufalin (Bu) and acetyl-bufalin (Ac-bu) for 48 h. Cell viability was determined via MTT assay, and IC_50_ values were calculated. **c** Human NSCLC cells were incubated with acetyl-bufalin for 24 h and allowed to form colonies for 1 week. Colonies were then fixed, stained with crystal violet and photographed. **d** PC-9 cells were plated in six-well plates for 24 h, then scratched and exposed to acetyl-bufalin for 48 h and observed microscopically at ×100 magnification. **e** The bufalin and acetyl-bufalin groups received an intraperitoneal injection at the dose of 30 mg/kg on the first day, and the mortality and weight of the mice were observed for 14 days. ****P < 0.0001. **f** Body weight curve of the mice. **g** Mean concentration-time curves of bufalin and acetyl-bufalin in rat plasma after administration intravenously at a single dose of 0.25 mg/kg (*n* = 3). **h** Mean concentration-time curves of bufalin and acetyl-bufalin in rat plasma after administration intraperitoneally at a single dose of 2 mg/kg (*n* = 3).

To solve the metabolic deficiency of bufalin and develop new analogues with higher efficiency and lower toxicity, the prodrug of bufalin, acetyl-bufalin was designed and synthesised (Fig. 1a and Supplementary Fig. S1). To investigate the effects of bufalin and acetyl-bufalin on human NSCLC cell lines, cell viability was evaluated via MTT assay. The IC_50_ values of bufalin and acetyl-bufalin were 140.9 nM and 64.04 nM, respectively, in PC-9 cells (Fig. 1b), indicating that the inhibitory effect of acetyl-bufalin was approximately twice that of bufalin. Two human NSCLC cell lines, A549 and H460, were selected to observe the effect of acetyl-bufalin on NSCLC cell proliferation. The IC_50_ values were 28.11 nM and 27.83 nM, respectively (Fig. 1b). Further, we choose two normal cells, human umbilical vein endothelial cell HUVEC and normal lung cells BEAS-2B, to analyse the cytotoxic effect of acetyl-bufalin with MTT assay. As shown in Supplementary Fig. S2a, acetyl-bufalin is low toxicity on normal cells compared with cancer cells. The effect of acetyl-bufalin on the cloning ability of NSCLC cells and normal cells was also explored. The colony-formation results showed that the NSCLC cell cloning ability was inhibited dose-dependently compared with normal cells (Fig. 1c and Supplementary Fig. S2b). To determine whether acetyl-bufalin could inhibit NSCLC cell migration, we performed a wound-healing assay using highly invasive PC-9 cells. Acetyl-bufalin markedly blocked PC-9 cell migration dose-dependently (Fig. 1d). Acute toxicity studies were performed to establish the safety of the compounds. The mice in the bufalin group (10/10) died immediately after the intraperitoneal injection of 30 mg/kg bufalin, while only 2/10 mice died in the acetyl-bufalin group (Fig. 1e). Compared with the vehicle group, the weight of the remaining mice in the acetyl-bufalin group was stable (Fig. 1f). Then we established a highly sensitive and reliable LC-MS/MS method and analysed the rat plasma pharmacokinetics of bufalin and acetyl-bufalin after administration. After bufalin administrated to rats, bufalin showed fast absorption with the large maximum drug concentration (Cmax) and a short time to reach maximum drug concentration (tmax). While after acetyl-bufalin administrated to rats, the plasma concentrations of acetyl-bufalin and bufalin, which were produced by metabolism of acetyl-bufalin, were relatively lower with smaller Cmax. However, the elimination of acetyl-bufalin and bufalin, which was produced by the metabolism of acetyl-bufalin in blood, was much slower than bufalin (Fig. 1g, h).

### Acetyl-bufalin-induced apoptosis in human NSCLC cells

**Fig. 2:**
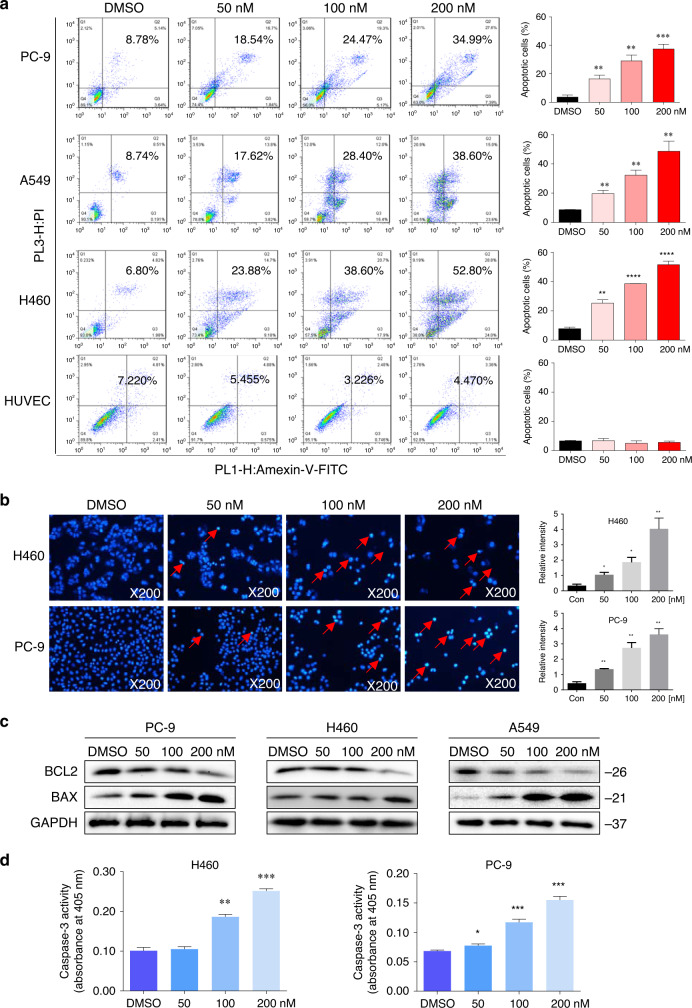
Acetyl-bufalin-induced apoptosis in human non-small-cell lung cancer (NSCLC) cells. **a** Human H460, PC-9, A549 and HUVEC cells were treated with different concentrations of acetyl-bufalin for 24 h, and the percentage of apoptotic cells was assessed using flow cytometry. Assays were performed in triplicate. **b** H460 and PC-9 cells were stained with Hoechst 33342 after 24 h of treatment with acetyl-bufalin and then observed under inverted fluorescence microscopy. **c** BCL2 and BAX expressions in H460, PC-9 and A549 cells were measured via western blot analysis after 24 h of treatment with acetyl-bufalin. **d** H460 and PC-9 cells were treated with different concentrations of acetyl-bufalin for 24 h then the activity of caspase-3 was determined. *P < 0.05, **P < 0.01, ***P < 0.001, ****P < 0.0001.

To evaluate the apoptosis-inducing effects of acetyl-bufalin in human NSCLC cells, human NSCLC cells were treated with acetyl-bufalin for 24 h, then stained with Annexin V-FITC and PI, and apoptotic cells were evaluated via flow cytometry. The data showed that acetyl-bufalin dose-dependently induced cell apoptosis in human NSCLC cells but did not affect normal human cells (Fig. 2a). Furthermore, the morphological features of apoptotic cells were visualised using Hoechst 33342 staining. Acetyl-bufalin was used to treat H460 and PC-9 cells for 24 h; then the cells were stained with Hoechst 33342. The percentage of acetyl-bufalin-induced apoptotic cells was significant compared with the untreated vehicle group (Fig. 2b). In addition, western blot analysis and caspase-3 activity assay further revealed that acetyl-bufalin dose-dependently increased the expressions of the proapoptotic proteins, BAX and caspase-3 activity, and decreased the level of the anti-apoptotic protein, BCL2, in human NSCLC cells (Fig. 2c, d and Supplementary Fig. S2c).

### Acetyl-bufalin-induced G2/M cell cycle arrest and inhibited the STAT3 signalling pathway in human NSCLC cells

**Fig. 3:**
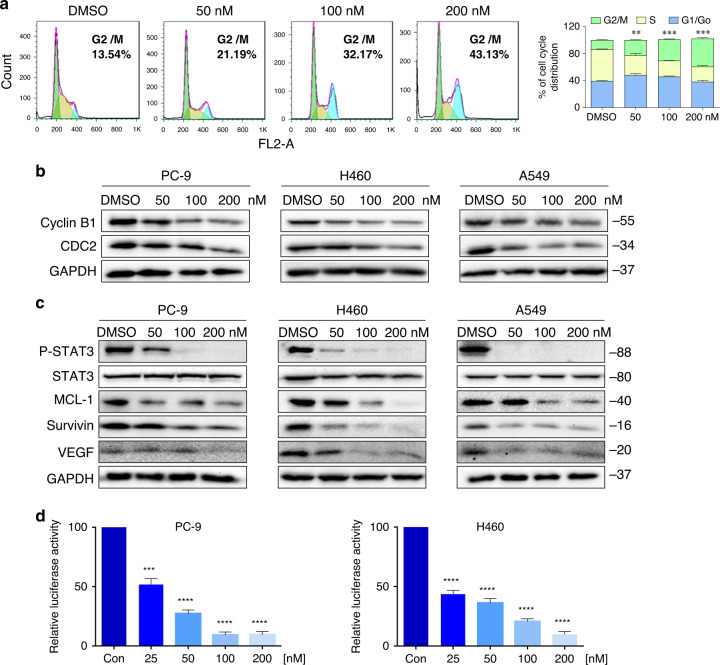
Acetyl-bufalin induced G2/M cell cycle arrest and inhibited the STAT3 signalling pathway in human non-small-cell lung cancer (NSCLC) cells. **a** Cell cycle distribution in PC-9 cells exposed to acetyl-bufalin was assessed via flow cytometry. Histogram of cell cycle distribution in PC-9 cells with and without acetyl-bufalin treatment. **b** Human NSCLC cells were treated with four concentrations of acetyl-bufalin (0, 50, 100, 200 nM). Western blot analysis was used to detect the levels of cyclin B1 and CDC2 after acetyl-bufalin treatment. **c** H460, A549 and PC-9 cells were treated with concentration gradients of acetyl-bufalin. STAT3 and its downstream target genes, including MCL-1, Survivin and VEGF, were detected via western blot analysis. **d** PC-9 and H460 cells were transfected with luciferase reporter gene plasmid and treated with acetyl-bufalin (0, 25, 50, 100, 200 nM) for 12 h. The results were normalised to the Renilla luciferase activity. **P < 0.01, ***P < 0.001, ****P < 0.0001.

To assess the cell cycle arrest associated with acetyl-bufalin, PC-9 cell lines were treated with acetyl-bufalin for 12 h, and the percentages of acetyl-bufalin-treated and untreated PC-9 cells in the G2/M phase were analysed via flow cytometry. Compared with the untreated vehicle group, acetyl-bufalin significantly induced the arrest of PC-9 cells in the G2/M phase of the cell cycle (Fig. 3a), followed by decreased cell growth and increased apoptosis. Western blot analysis indicated that treatment with acetyl-bufalin dose-dependently inhibited the expressions of cyclin B1 and CDC2 in human NSCLC cells (Fig. 3b and Supplementary Fig. S2d). Thus, the inhibitory effect of acetyl-bufalin on cell proliferation is partly associated with inducing G2/M phase arrest in human NSCLC cells.

A derivative of bufalin has been reported to play an anti-tumour role by inhibiting the STAT3 pathway;^26^ thus, we thought that acetyl-bufalin may affect STAT3 phosphorylation (P-STAT3). We tested the inhibitory ability of acetyl-bufalin on STAT3 phosphorylation in human NSCLC cells. STAT3 phosphorylation was assessed at different levels of confluence, as in the previous study.^27^ As expected, acetyl-bufalin reduced the P-STAT3 protein levels in H460, A549 and PC-9 cells concentration-dependently after 12 h of treatment. STAT3 participates in oncogenesis by mediating its target genes, including BCL2, MCL-1, Survivin and VEGF.^28^ Analysis of the STAT3 target gene products revealed that acetyl-bufalin significantly reduced these target genes expression (Fig. 3c and Supplementary Fig. S3). In agreement with the data obtained using western blot assay, we found that acetyl-bufalin significantly inhibited P-STAT3 activation in a dose-dependent manner by using the luciferase reporter assay (Fig. 3d).

### Proteomic identification of acetyl-bufalin-binding proteins and molecular docking

**Fig. 4:**
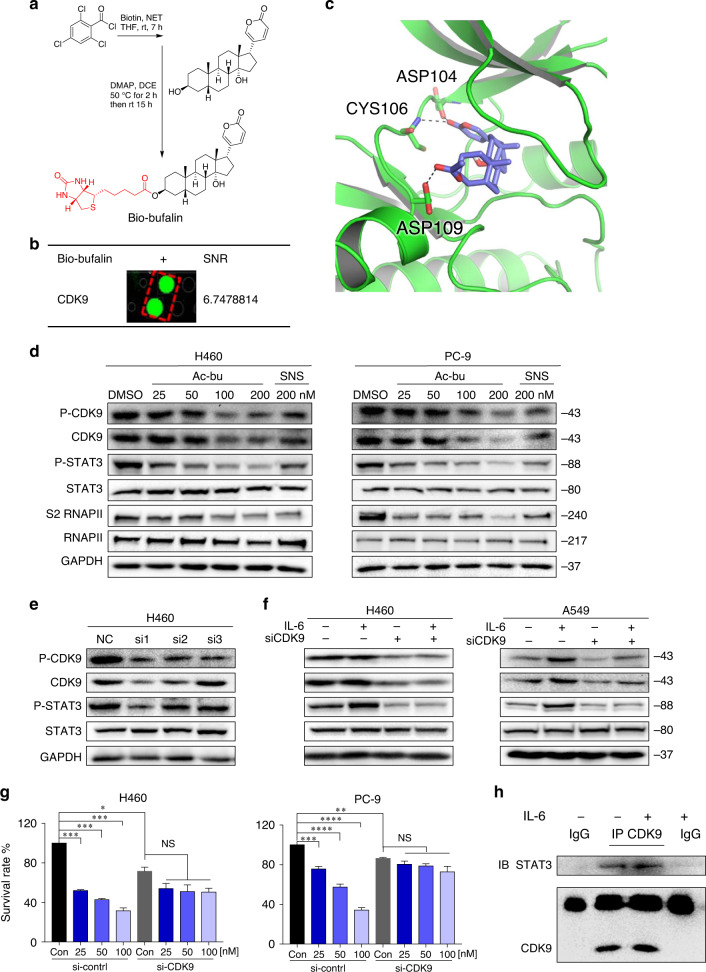
Acetyl-bufalin inhibited the CDK9/STAT3 signalling pathway. **a** Chemical structure of biotin-labelled bufalin (bio-bufalin). **b** Magnified image of bio-bufalin binding to recombinant CDK9 protein spots on the microarray; signal-to-noise ratio (SNR) is shown. **c** Molecular docking of acetyl-bufalin to the CDK9 binding spots. **d** The expression of proteins involved in the CDK9/STAT3 pathway in H460 and PC-9 cells was examined by western blotting after 12 h of acetyl-bufalin (Ac-bu) and SNS-032 (SNS) treatment. **e** CDK9 and STAT3 expression levels by western blotting 48 h post transduction with CDK9 siRNA (si1, si2 and si3) in H460 cells. **f** Western blot analysis of CDK9 and STAT3 expression levels in H460 and PC-9 cells transfected with CDK9 siRNA1 and treated for 30 min with or without IL6 (25 ng/ml). The data are representative of three independent experiments. **g** Relative quantification of cell proliferation in H460 and PC-9 cells transfected with CDK9 siRNA1 and treated with a concentration gradient of acetyl-bufalin for 48 h. **h** Co-immunoprecipitation analysis of STAT3 and CDK9 in H460 cells treated with IL6 (25 ng/ml). *P < 0.05, **P < 0.01, ***P < 0.001, ****P < 0.0001.

Based on previous studies,^26^ we speculated that STAT3 might be a direct target of acetyl-bufalin. To confirm our hypothesis, we screened for potential acetyl-bufalin-binding proteins. We first generated biotinylated bufalin (bio-bufalin; Fig. 4a and Supplementary Fig. S4). To ensure that the generated bio-bufalin retained its inhibitory activity in human NSCLC cells, we evaluated cell viability and compared the results with those of acetyl-bufalin. The IC_50_ value of bio-bufalin in PC-9 cells was 77.07 nM, which was similar to the IC_50_ (64.20 nM) of acetyl-bufalin (Supplementary Fig. S5a). Therefore, bio-bufalin was selected for subsequent protein microarray study. We then used bio-bufalin in a human proteomic microarray containing 19,394 affinity-purified N-terminal GST-tagged proteins covering ~75% of the human proteome. Briefly, microarrays were probed with bio-bufalin, and binding was detected with a Cy3-conjugated streptavidin (Cy3-SA). We then calculated the SNR of each spot, which was defined as the ratio of the median foreground minus the median background. No STAT3 was found in 35 positive proteins with SNR values greater than 1.5, suggesting that bio-bufalin may not be a direct target of STAT3. However, CDK9 bound to bio-bufalin in 35 positive proteins, with the strongest SNR being 6.7478814 (Fig. 4b and Supplementary Fig. S5b). In addition, the docking result revealed that acetyl-bufalin occupied the ATP-binding site and formed three hydrogen bonds with residues ASP104, CYS106 and ASP109 (Fig. 4c). Thus, acetyl-bufalin may be an ATP-competitive inhibitor. Based on the HuProt^TM^ human proteome microarray and docking results, we speculated that CDK9 may be a direct target of acetyl-bufalin.

### Acetyl-bufalin inhibited the CDK9/STAT3 signalling pathway

To investigate the CDK9 signalling pathway, we measured the expressions of several downstream target proteins of CDK9 after acetyl-bufalin treatment. After incubating H460 and PC-9 cells with 25, 50, 100, or 200 nM acetyl-bufalin and 200 nM SNS-032 (a known CDK9 inhibitor) for 12 h, P-CDK9, CDK9, P-STAT3 and S2 RNAPII expressions were significantly dose-dependently decreased, whereas the total STAT3 and RNAPII expressions were not significantly changed (Fig. 4d and Supplementary Fig. S5c). IL6 is the main inducer of the STAT3 pathway in NSCLC and promotes tumour growth. CDK9 promoted IL6-induced STAT3 phosphorylation; conversely, CDK9 inhibition by siCDK9 blocked IL6-induced STAT3 phosphorylation dose-dependently (Fig. 4e, f and Supplementary Fig. S6a, b). To confirm the ability of acetyl-bufalin to reduce cell proliferation and test whether this effect was CDK9-dependent, we examined the inhibitory effect of acetyl-bufalin on the proliferation of H460 and PC-9 cells depleted or not of CDK9. Acetyl-bufalin did not significantly affect the siCDK9 cells. Conversely, in cells transfected with a control siRNA (and therefore expressing CDK9), acetyl-bufalin hampered cell proliferation in H460 and PC-9 cells (Fig. 4g). These results strongly suggest that CDK9 is a direct target of acetyl-bufalin.

Next, we determined whether CDK9 interacted with STAT3 in human NSCLC cells, which was confirmed via co-immunoprecipitation experiments. H460 cells were stimulated with IL6 for 30 min or left untreated, whole-cell extracts were recovered, and coimmunoprecipitations were performed with polyclonal antibodies directed against CDK9, STAT3 or nonspecific antibodies. Under these conditions, CDK9 was coimmunoprecipitated with STAT3 proteins (Fig. 4h). These coimmunoprecipitations were carried out without using transfected cells so that the association did not require overexpressing the proteins. These results indicate that STAT3 interacts, likely directly, with CDK9.

### Acetyl-bufalin inhibited NSCLC xenograft model growth

**Fig. 5:**
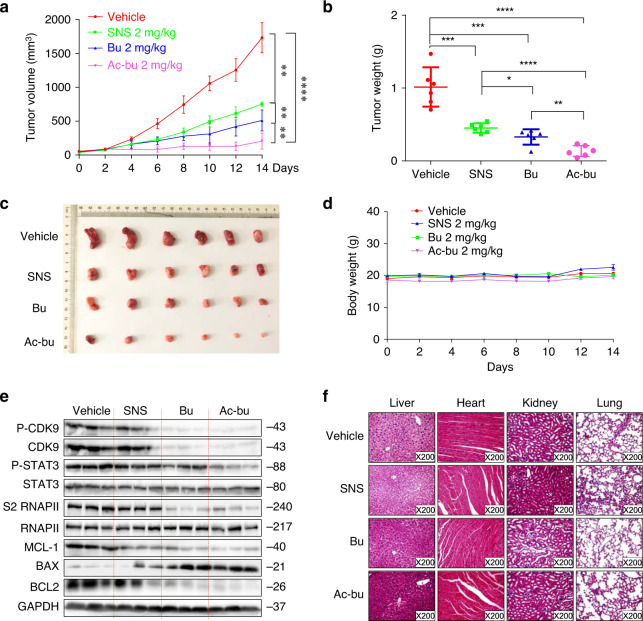
Acetyl-bufalin inhibited growth of non-small-cell lung cancer (NSCLC) xenograft models. **a**, **b** Volume and weight of tumour tissue in anatomical nude mice treated with SNS-032(SNS), bufalin (Bu) or acetyl-bufalin (Ac-bu) (2 mg/kg). **c** Tumour tissue of nude mice treated with SNS-032(SNS), bufalin (Bu) or acetyl-bufalin (Ac-bu) (2 mg/kg). **d** Nude mouse size growth curve. **e** Western blot analysis on the expressions of CDK9/STAT3 pathway-associated proteins and apoptosis-related proteins in excised tumour tissue lysates. **f** Haematoxylin and eosin staining of hearts, livers, lungs and kidneys from the nude mice at ×200 magnification. *P < 0.05, **P < 0.01, ***P < 0.001, ****P < 0.0001.

Because of the potent cytotoxic activity of acetyl-bufalin on human NSCLC cells, we next assessed the therapeutic efficacy of acetyl-bufalin in H460 xenograft models. Mice were intraperitoneally injected with 2 mg/kg SNS-032, bufalin or acetyl-bufalin every other day. The tumour weight and volume were measured, and SNS-032, bufalin and acetyl-bufalin significantly inhibited tumour growth compared with that of the vehicle group. Acetyl-bufalin had the best inhibitory effect (Fig. 5a–c). Importantly, the body weights of the mice remained stable (Fig. 5d). Remarkably, P-CDK9, P-STAT3 and S2 RNAPII expressions were decreased significantly after acetyl-bufalin treatment. In addition, acetyl-bufalin treatment markedly increased apoptosis as indicated by the BAX and BCL2 expressions (Fig. 5e) and had no gross toxicities on the hearts, livers, kidneys or lungs compared with those of the vehicle group (Fig. 5f), demonstrating excellent safety profiles.

### Anti-tumour activity of acetyl-bufalin in a PDX animal model of NSCLC

**Fig. 6:**
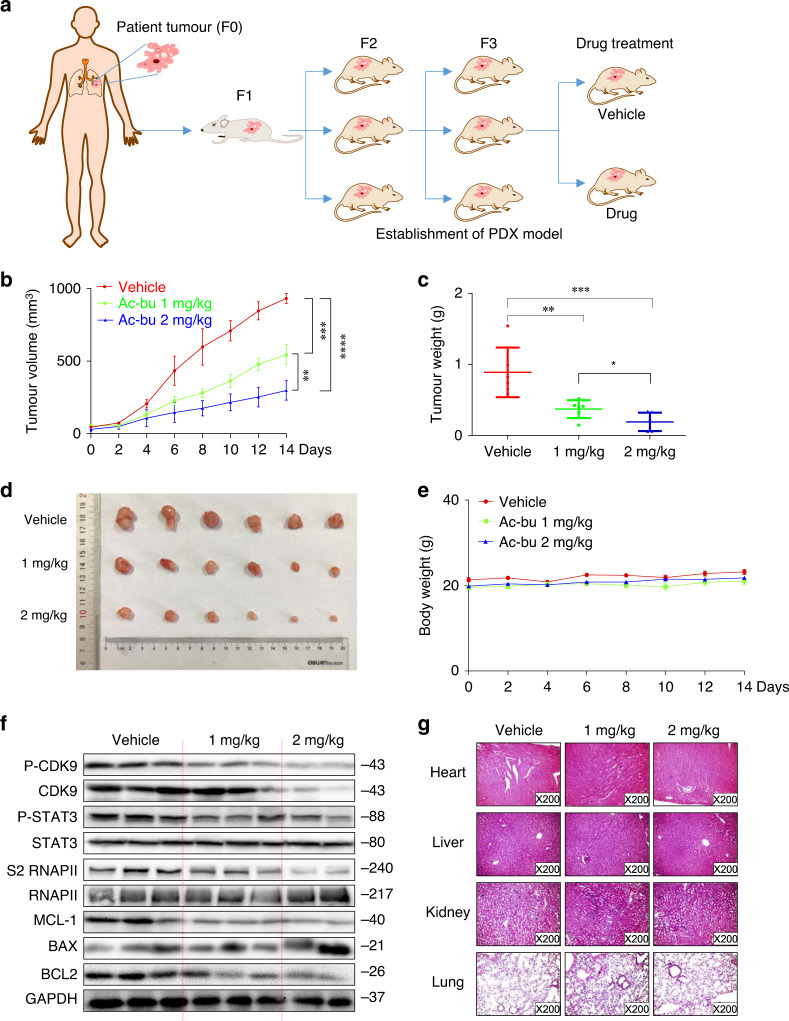
Anti-tumour activity of acetyl-bufalin in a patient-derived (PDX) non-small-cell lung cancer (NSCLC) xenograft animal model. **a** Flowchart for establishing the PDX model. **b** Tumour volume in the PDX model. **c** Curve of the tumour weights in the mice. **d** Images of representative tumours removed from the PDX model. **e** Body weight curve of the PDX-model nude mice. **f** Western blot analysis on the expressions of CDK9/STAT3 pathway-associated proteins and apoptosis-related proteins from tumour tissue removed from the PDX mouse model. **g** Haematoxylin and eosin staining of hearts, livers, lungs and kidneys from the nude mice at ×200 magnification. *P < 0.05, **P < 0.01, ***P < 0.001, ****P < 0.0001.

PDX animal models may be superior to standard cell-line xenograft models of cancer because they are more similar to the parental tumours. PDX models are attractive choices for testing novel compounds. Four NSCLC specimens and their corresponding adjacent normal tissues were obtained surgically after primary diagnosis. To establish the PDX animal model, we designed a procedure, as shown in Fig. 6a. Of the four samples, only one passable xenograft model was successfully established. Third-generation mice were randomly divided into three groups. Tumour growth was obviously decreased in the acetyl-bufalin-treated group (Fig. 6b). The average volumes of the tumours treated with acetyl-bufalin were 544.43 mm^3^ (1 mg/kg per 2 days) and 256.29 mm^3^ (2 mg/kg per 2 days). However, the average volume of the vehicle group tumours was 931.90 mm^3^ (Fig. 6b). Acetyl-bufalin led to a supra-additive reduction in growth in the xenograft models (Fig. 6c, d), and the body weight of the mice was stable (Fig. 6e). Notably, P-CDK9 and CDK9 expressions were downregulated after acetyl-bufalin treatment, indicating that CDK9 remained a major target of acetyl-bufalin in vivo (Fig. 6f). In addition, compared with the other three groups, acetyl-bufalin significantly decreased BCL2 levels and increased BAX levels. Moreover, the acetyl-bufalin treatment therapy had no gross toxicities on the heart, liver, kidney and lung compared with the vehicle group (Fig. 6g), demonstrating excellent safety profiles. These data indicated that the anti-tumour effects of acetyl-bufalin were due to inhibition of CDK9/STAT3 in the PDX animal model and supported that acetyl-bufalin may display potential therapeutic effects in clinical trials.

### CDK9 is highly expressed in human NSCLC tissues

To assess the roles of CDK9 in human NSCLC, we first evaluated CDK9 protein expression in 12 human NSCLC tissues and their paired adjacent tissues. The CDK9 protein level was significantly upregulated in human NSCLC specimens compared with that of normal lung tissues. CDK9 was increased in 10 of the 12 tumour tissues (83.33%) compared with that in the adjacent tissues (Supplementary Fig. S7a). To further confirm this result, we surveyed the CDK9 protein expression via immunohistochemistry. Immunohistochemistry analysis also showed that CDK9 was highly expressed in most human NSCLC tissues (Supplementary Fig. S7b). Collectively, our data suggest that CDK9 is significantly upregulated in NSCLC. Thus, CDK9 may serve as a novel diagnostic marker of NSCLC.

## Discussion

Enhanced circulation is the critical factor for promoting desired and acceptable pharmacokinetic behaviour leading to more delivery to the tumour site and less side effects.^29,30^ In vivo administration acetyl-bufalin led to the release of bufalin, which improved the pharmacokinetics of bufalin (Fig. 1g, h). Meanwhile, acetyl-bufalin was better tolerated in mice at a high-dose-level treatment than bufalin was (Fig. 1e, f). All these results demonstrated that acetyl-bufalin could change the pharmacokinetic characteristics of bufalin and prolong the action time, which means that it can reduce the times of administration and reduce the toxic and side effects caused by repeated bufalin administration. Instead, acetyl-bufalin has a safety profile that can be achieved by long intravenous infusions.

CDK9 inhibitors have been investigated as therapeutics for various haematological cancers and solid tumours.^31–33^ Several CDK9 inhibitors, such as AZD4573, BAY 1143572, FIT-039, THAL-SNS-032 and JSH-150, have been successfully developed and have shown promising efficacy in preclinical and clinical studies.^19,32,34–36^ To date, the activity observed does not match the initial expectations for CDK9 inhibitors, and no CDK9 inhibitor has been approved for clinical use,^37,38^ thus far, necessitating investigating more targeted approaches to improve outcomes. The optimal pharmacokinetic profile and dosing schedule for CDK9 inhibitors remain to be determined.^20,37^ In this study, acetyl-bufalin, a prodrug of bufalin, was designed and synthesised with higher efficiency and lower toxicity. Acetyl-bufalin can exert anti-tumour effect by inhibiting the CDK9/STAT3 signalling pathway (Fig. 4). Bufalin, as the main active constituent of chansu or Huachansu/cinobufacin, are already in use to treat patients, based on observed pharmacological activities, including anti-tumour, immunomodulation and attenuation of cancer-derived pain,^11,39^ but has adverse effects when administered as a single agent.^12,14^ Ideally, acetyl-bufalin is predicted to have a noncytotoxic mechanism of action towards noncancerous tissues (Figs. 5 and 6), and its pharmacokinetic profile should provide sufficient exposure throughout the whole-cell cycle with a reasonable administration schedule. However, the future anti-tumour research on acetyl-bufalin (and its analogues) needs to follow the standard pharmacology guidelines to ensure the efficacy and safety of chansu-derived substances based on evaluation of pharmacodynamic-related and toxicity-related issues.

CDK9 is usually associated with transcriptional elongation, which can directly modulate translation through co-opting components of the major translational regulatory complex.^18^ Recent studies have identified other cellular factors that interact with CDK9, including mTOR, BRD4 and Hsp90. Two distinct complexes of CDK9 and mTOR (CTORC1 and CTORC2) play key roles in mRNA transcription and translation of mitogenic genes.^40^ Complex transcriptional elongation of BRD4 and CDK9 is necessary for TGFβ-induced Nox4 expression and myofibroblast transdifferentiation.^41^ The Hsp90/Cdc37/CDK9 complex also exists in resting T cells lacking cyclin T1.^42^ Sirtuin 2 (SIRT2)-mediated deacetylation of CDK9 deacetylation is essential for STAT1 phosphorylation at Ser-727.^43^ The STAT3-CDK9 complex can stimulate γ-FBG gene recruitment and prolong transcription. IL6 can increase both CDK9 and cyclin T1 protein levels in peripheral blood lymphocytes.^21^ STAT3 promotes transcription initiation and regulates chromatin remodelling and transcription elongation by interacting with BRG1 and CDK9.^44^ STAT3 phosphorylation occurs upon promoter binding by an unknown kinase.^45,46^ In our study, transient transfection assays revealed that CDK9 activity was required to activate IL6-induced STAT3, and western blotting showed that CDK9 knockdown significantly suppressed STAT3 expression. CDK9 was induced in response to IL6, which could be another mechanism for regulating STAT3 activity.

We demonstrate that the activity of CDK9 is necessary to maintain the transcription process induced by STAT3 proteins, which is activated abnormally by mutation and extracellular signal. MCL-1 and BCL2 are among the downstream genes transcriptionally regulated by the CDK9/STAT3 axis. It will be a significant research direction to determine whether CDK9 has a general mechanism to regulate some or all STAT3-dependent genes by detecting the kinetics of CDK9 association on different STAT3 target gene promoters through ChIP assays. Further studies of other STAT3 target genes may answer the question of whether all STAT3-dependent genes generally need CDK9 activity. Our findings that STAT3 phosphorylation is changed by CDK9 inhibition indicates that STAT3 may be a target of CDK9. Acetyl-bufalin reduces CDK9/RNA polymerase serine 2-mediated STAT3 transcription activity across representative NSCLC cell lines. Moreover, our data show that acetyl-bufalin treatment specifically and efficiently suppresses CDK9-STAT3 formation. This mechanism may be used to develop a novel therapeutic combination of acetyl-bufalin in NSCLC preclinical models. CDK9 is a novel prognostic marker and therapeutic target in ovarian cancer, osteosarcoma and leukaemia.^16,36,47^ Our findings also suggest that CDK9 may serve as a novel diagnostic marker of NSCLC.

## Conclusions

It is conceivable that acetyl-bufalin can be further explored as a unique CDK9 inhibitor for anti-tumour therapy. Future studies will confirm the role of CDK9 in tumour metastasis and chemoresistance and explore the synergistic effects of acetyl-bufalin in combination with other chemotherapies.

## Supplementary information

Supplementary files

## Data Availability

All data and material presented in this article and in the Supplementary Information are available upon request from the corresponding author.
