# Supplementary material for: Acetyl-bufalin shows potent efficacy against non-small-cell lung cancer by targeting the CDK9/STAT3 signalling pathway
Source: Br J Cancer. 2020 Oct 30;124(3):645–57. doi: 10.1038/s41416-020-01135-6 (PMC7851395; doi:10.1038/s41416-020-01135-6)
Supplement: Supplementary file 1 — Supplementary files [file 41416_2020_1135_MOESM1_ESM.pdf]

Figure S1

A

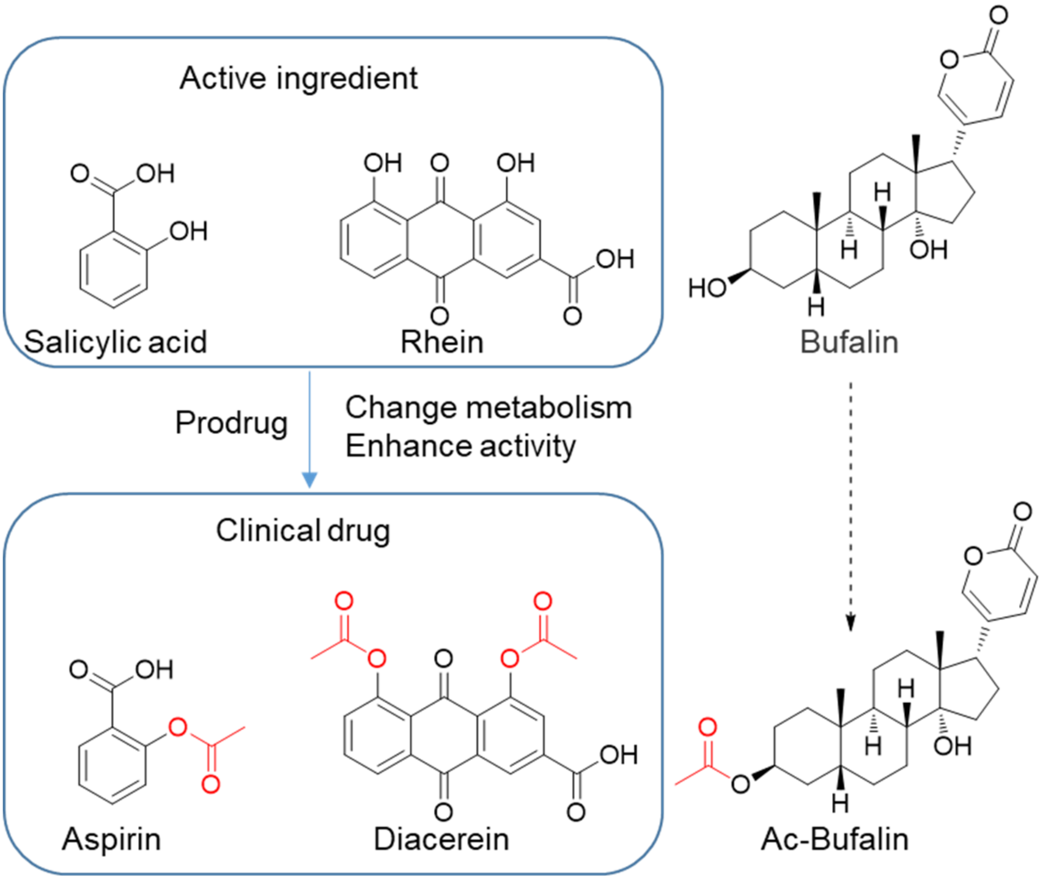

Active ingredient
Salicylic acid
Rhein
Bufalin
Prodrug
Change metabolism
Enhance activity
Clinical drug
Aspirin
Diacerein
Ac-Bufalin

B

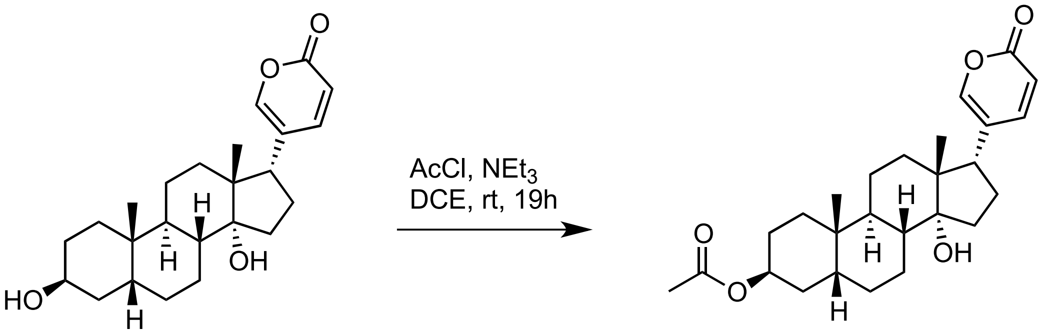

AcCl, NEt3
DCE, rt, 19h

Figure S2

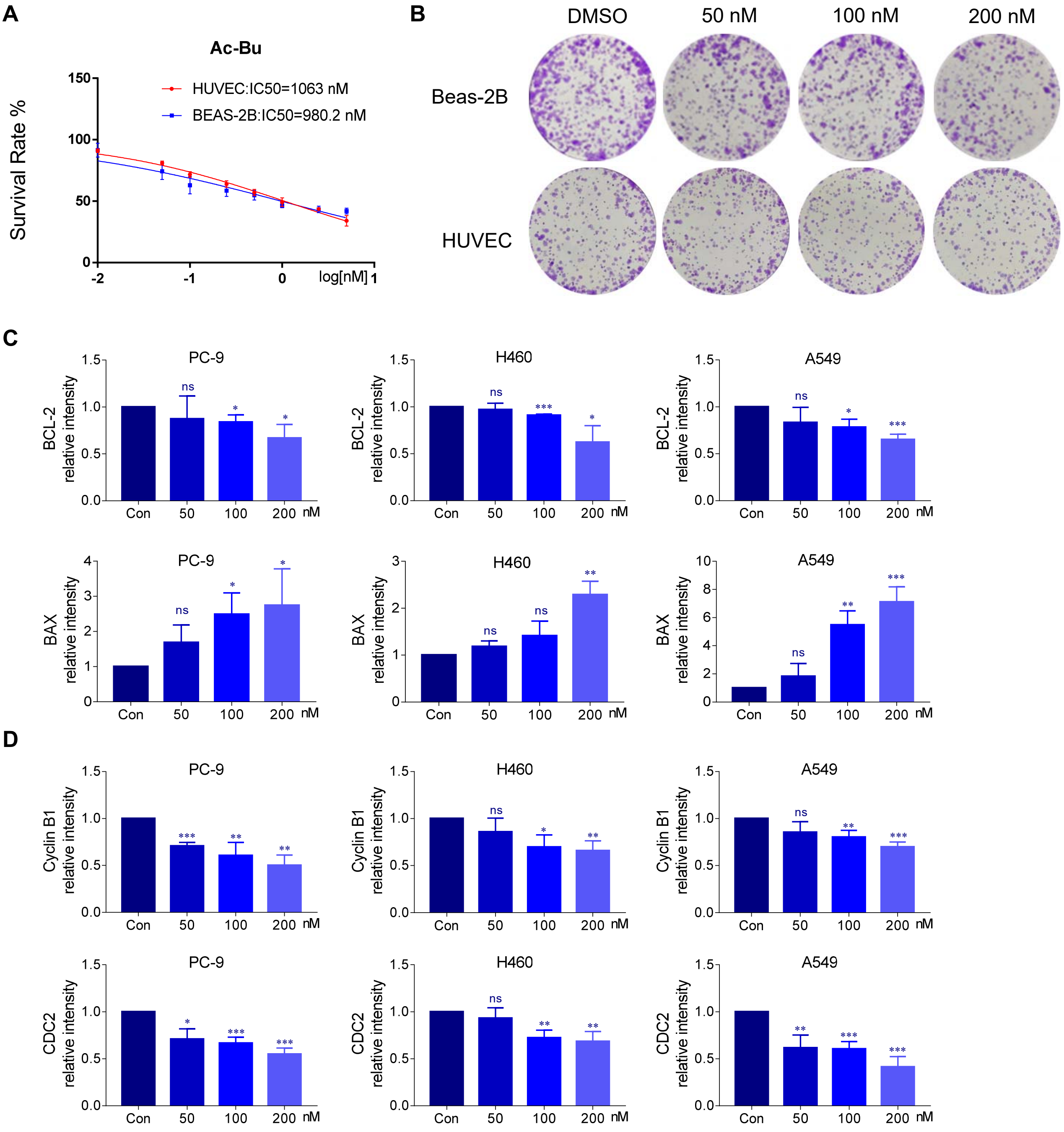
A
Ac-Bu
HUVEC:IC50=1063 nM
BEAS-2B:IC50=980.2 nM
Survival Rate %
log[nM]
B
DMSO
50 nM
100 nM
200 nM
Beas-2B
HUVEC
C
PC-9
H460
A549
BCL-2
relative intensity
BAX
relative intensity
Con
50
100
200
nM
D
Cyclin B1
relative intensity
CDC2
relative intensity

Figure S3

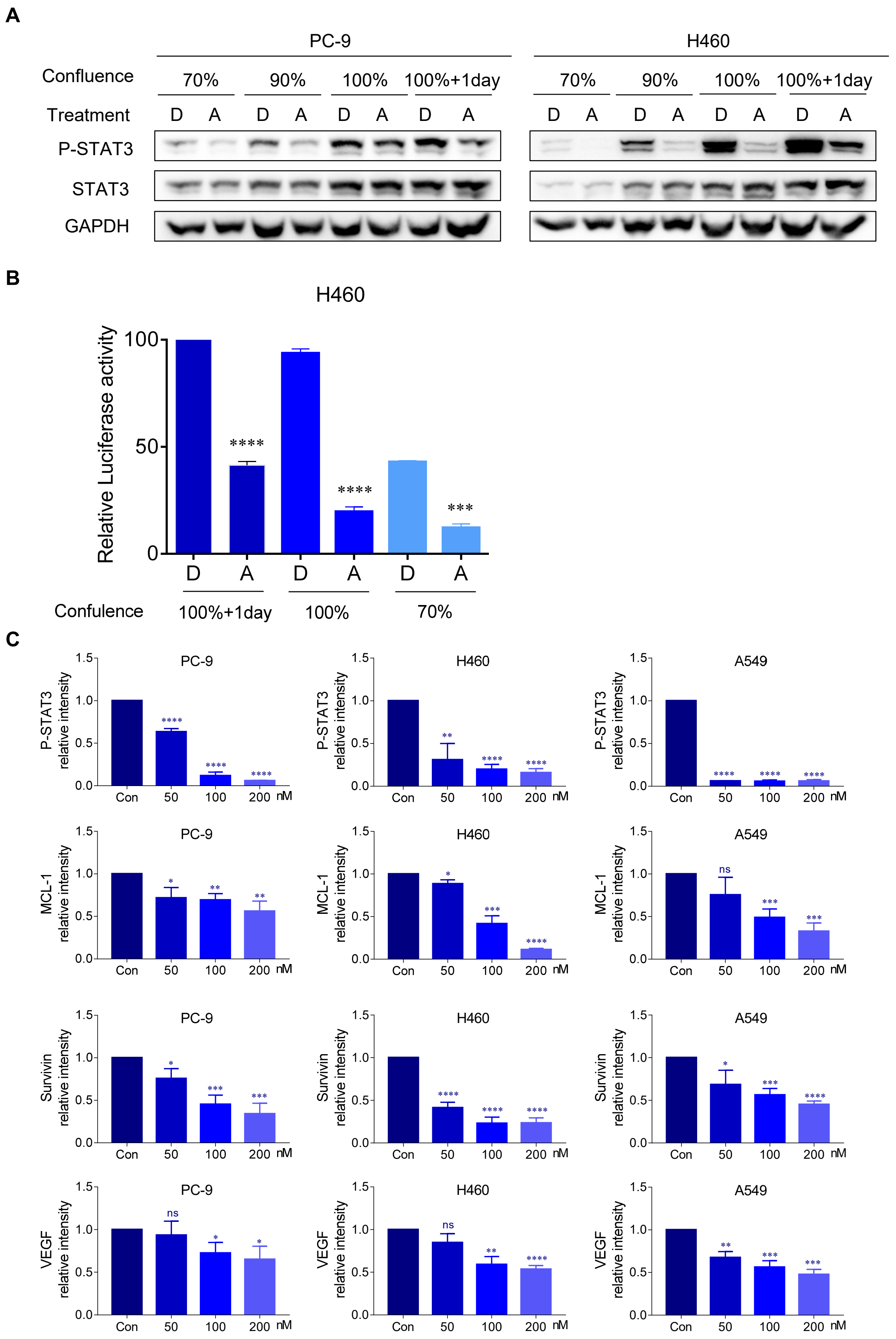

A
PC-9
H460
Confluence
70%
90%
100%
100%+1day
Treatment
D A D A D A D A
P-STAT3
STAT3
GAPDH
B
H460
Relative Luciferase activity
100
50
0
****
****
***
D A D A D A
Confluence
100%+1day
100%
70%
C
PC-9
H460
A549
P-STAT3 relative intensity
MCL-1 relative intensity
Survivin relative intensity
VEGF relative intensity
Con 50 100 200 nM

Figure S4

A

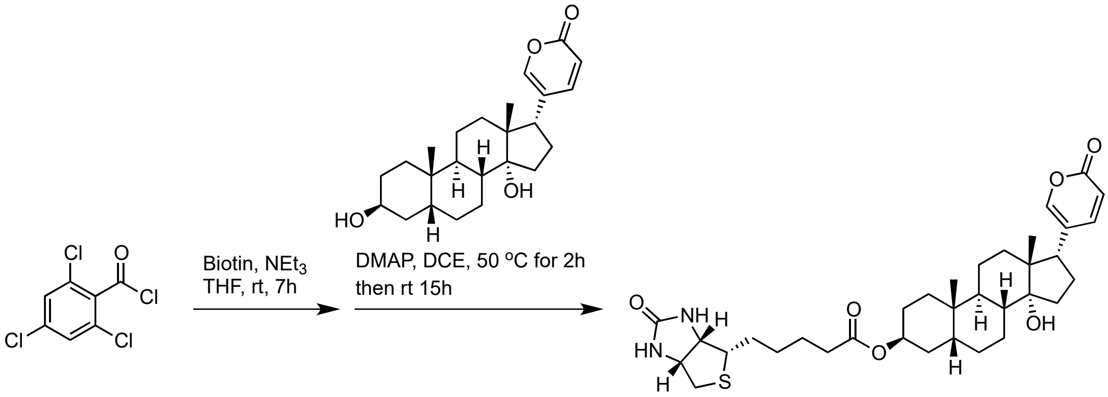

B

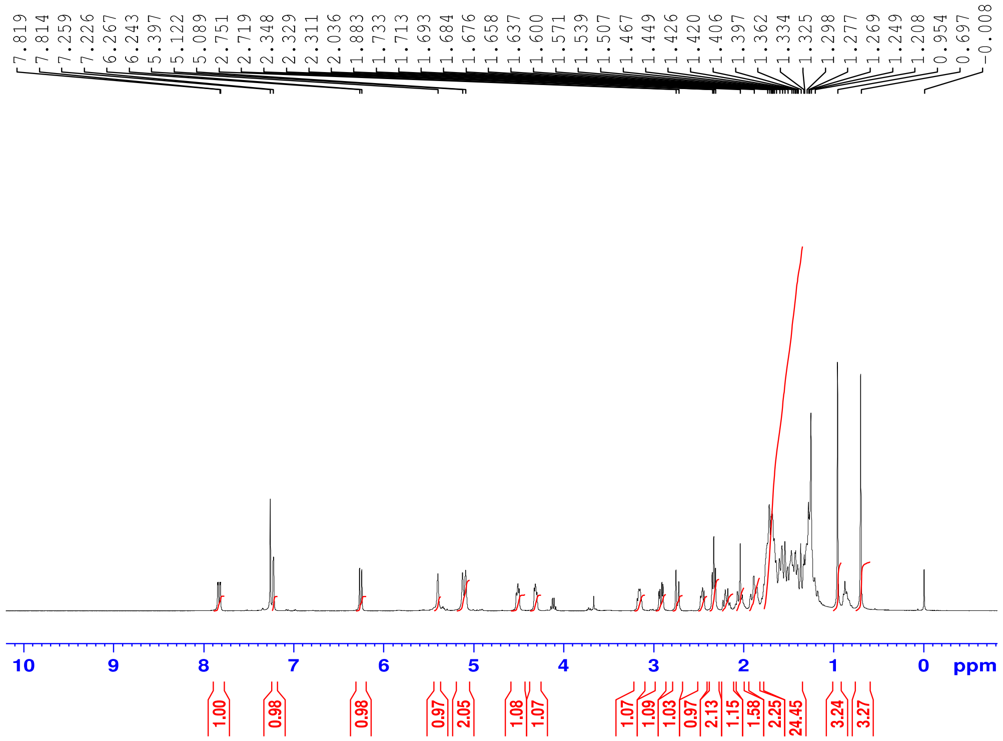

C

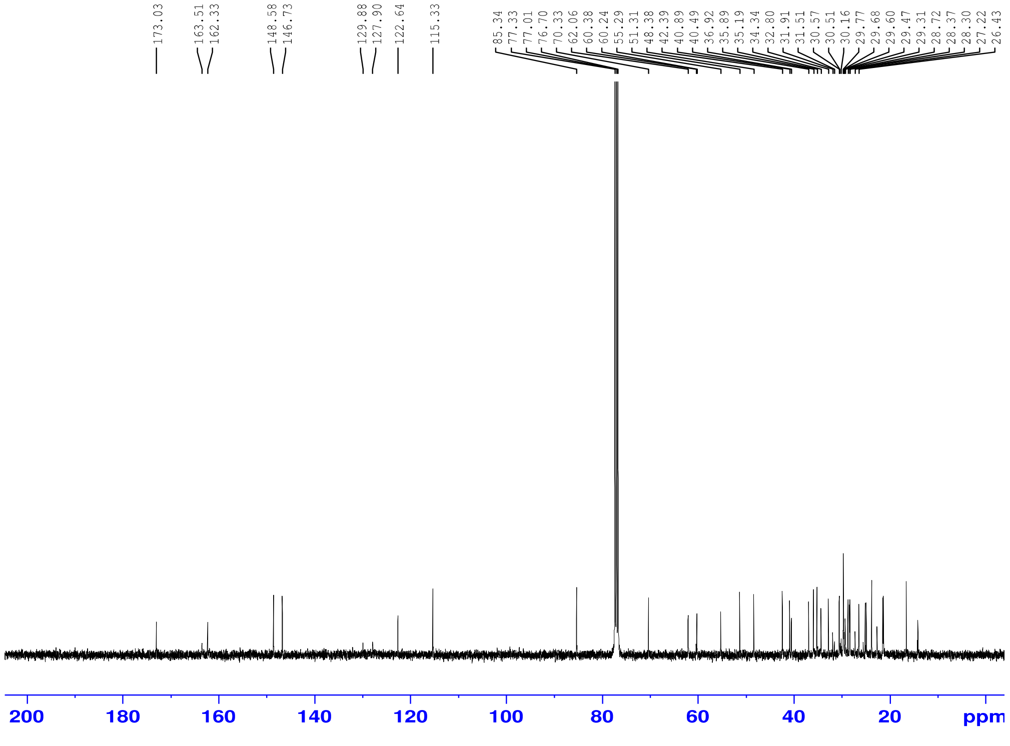

Figure S5

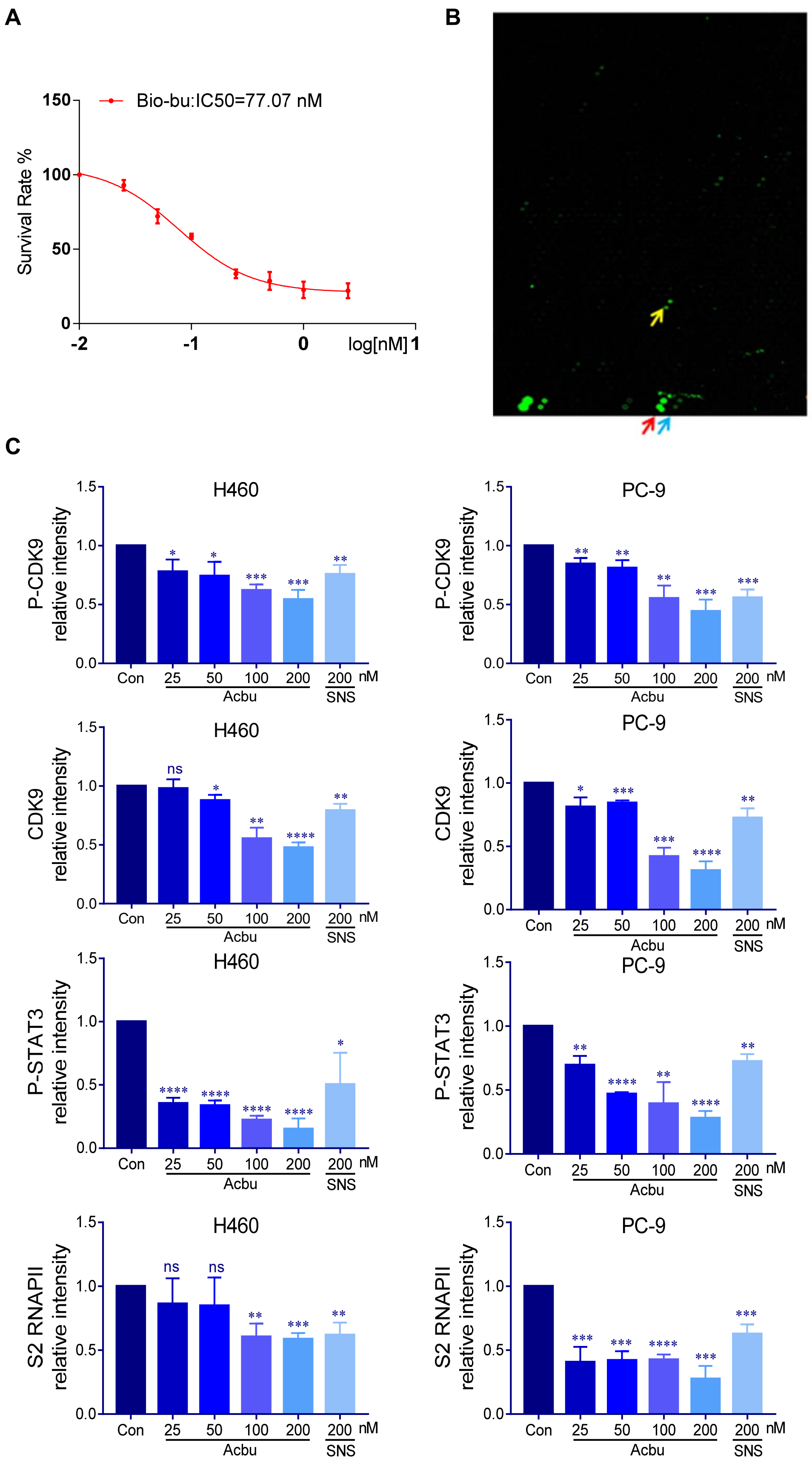
A
B
C
Bio-bu:IC50=77.07 nM
Survival Rate %
log[nM]
H460
PC-9
P-CDK9 relative intensity
CDK9 relative intensity
P-STAT3 relative intensity
S2 RNAPII relative intensity
Con
25
50
100
200
200
nM
Acbu
SNS

Figure S6

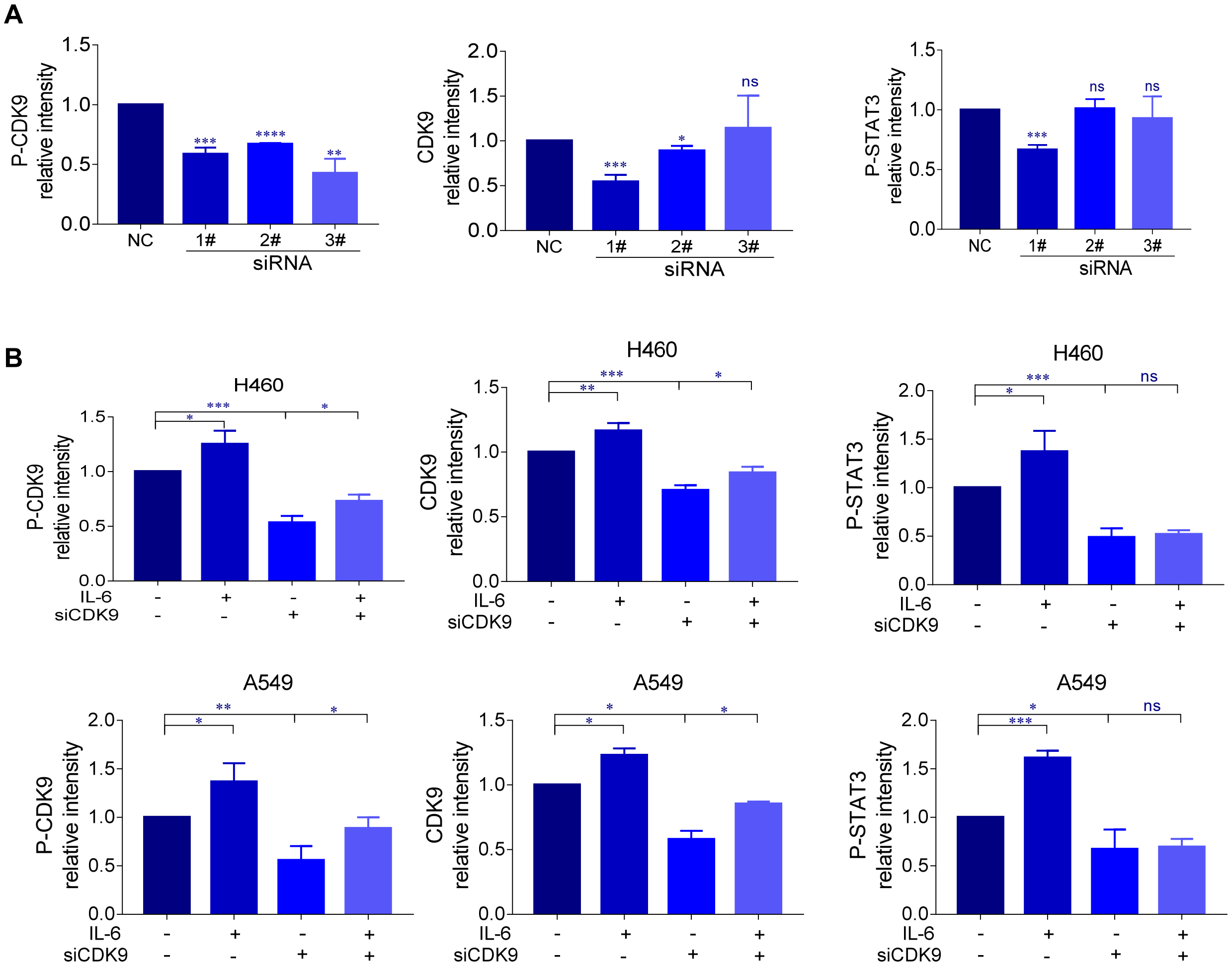
A
P-CDK9 relative intensity
CDK9 relative intensity
P-STAT3 relative intensity
NC
1#
2#
3#
siRNA
ns
B
H460
A549
P-CDK9 relative intensity
CDK9 relative intensity
P-STAT3 relative intensity
IL-6
siCDK9
ns

Figure S7

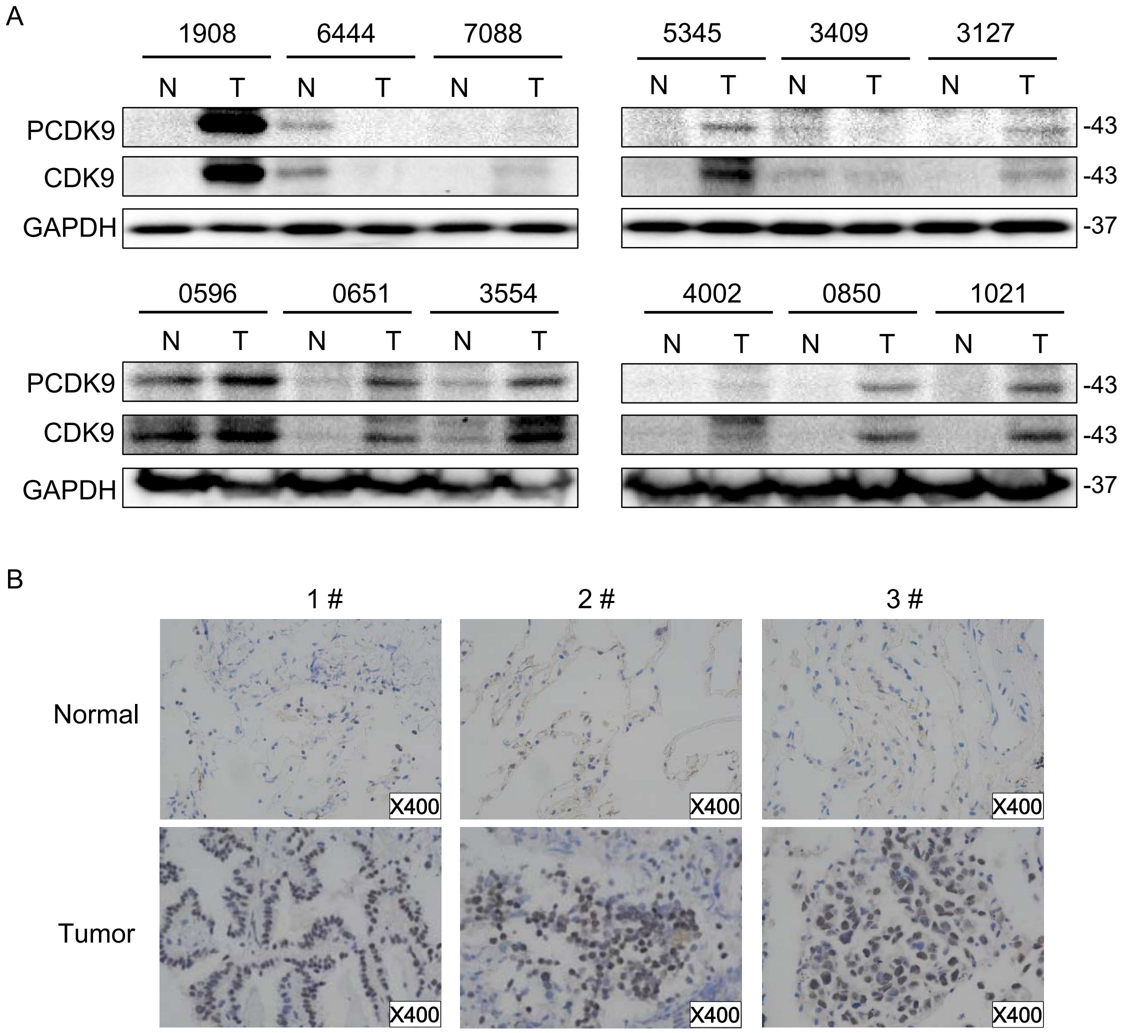
A
1908
6444
7088
5345
3409
3127
N T N T N T
N T N T N T
PCDK9
CDK9
GAPDH
-43
-43
-37
0596
0651
3554
4002
0850
1021
N T N T N T
N T N T N T
PCDK9
CDK9
GAPDH
-43
-43
-37
B
1 #
2 #
3 #
Normal
Tumor
X400

Fig. S1 Designed and synthesized acetyl-bufalin

A. Designed and synthesized acetyl-bufalin (Ac-bufalin) base on aspirin (acetylsalicylic acid) and diacerein (diacetoxy-rhein). **B.** Chemistry experimental section of Ac-bufalin: To a mixture of bufalin (15 mg, 0.0388 mmol) and NEt_3 (0.39 mmol) in DCE (dry, 1 mL) was added AcCl (0.39 mmol), the reaction mixture was stirred at rt for 19 h. The mixture was then purified by a silica gel flash chromatography (DCM/MeOH = 50:1 to 20:1) to afford target compound (17 mg, 100% yield) as a pale yellow solid. ^{1}H NMR (400 MHz, $CDCl_3$) δ 7.82 (dd, *J* = 9.6, 4.0 Hz, 1H), 7.22 (s, 1H), 6.26 (d, *J* = 9.6 Hz, 1H), 5.07 (s, 1H), 2.49-2.40 (m, 1H), 2.26-2.15 (m, 1H), 2.09-1.99 (m, 4H), 1.94-1.81 (m, 2H), 1.80-1.35 (m, 18H), 1.27 (s, 3H), 0.69 (s, 3H); ^{13}C NMR (100 MHz, $CDCl_3$) δ 170.7, 162.3, 148.6, 146.7, 122.6, 115.3, 85.4, 70.4, 51.3, 48.4, 42.4, 40.9, 36.8, 35.9, 35.2, 32.8, 30.5, 30.5, 28.7, 26.4, 25.1, 23.7, 21.5, 21.4, 21.3, 16.5; ESI-HRMS: calcd. for $C_{26}H_{37}O_5^+$ $(M+H)^+$ 429.2636, found 429.2637.

Fig. S2 Antitumor activity of acetyl-bufalin *in vitro*

A. HUVEC and BEAS-2B cells were incubated with increasing doses of acetyl-bufalin for 48 h. Cell viability was determined by the MTT assay. **B.** HUVEC and BEAS-2B cells were incubated with acetyl-bufalin for 24 h and allowed to form colonies for one week. Colonies were then fixed, stained with crystal violet and photographed. **C.** Cells were treated with acetyl-bufalin at different concentrations as indicated for 24 h, the cell lysates were processed for Western blot analysis for protein expression of BCL-2 and BAX, and the relative intensity was calculated. **D.** Western blot analysis was used to detect the levels of cyclin B1 and CDC2 after acetyl-bufalin treatment and the relative intensity was calculated. *P<0.05, **P<0.01, ***P<0.001.

Fig. S3 Acetyl-bufalin inhibited the STAT3 signaling pathway in human NSCLC cells

A. PC-9 and H460 cells were grown to 70, 90 or 100% confluence and up to 1 day postconfluence as indicated, in the presence of 50 nM acetyl-bufalin or the DMSO diluent. Cell lysates were used to detect P-STAT3, STAT3 and GAPDH as a control. **B.** H460 cells were grown to 70, or 100% confluence and up to 1 day postconfluence, then transfected with luciferase reporter gene plasmid and treated with 50 nM acetyl-bufalin or the DMSO diluent. The results were normalized to the

Renilla luciferase activity. **C.** H460, A549 and PC-9 cells treated with concentration gradients of acetyl-bufalin. STAT3 and its downstream target genes, including MCL-1, Survivin and VEGF, were detected via western blot analysis. The relative intensity was calculated. *P<0.05, **P<0.01, ***P<0.001, ****P<0.0001.

Fig.S4 Chemistry experimental section of Bio-bufalin

To a mixture of Biotin (0.3 mmol) and NEt_3 (0.45 mmol) in THF (dry, 3 mL) was added 2,4,6-trichlorobenzoyl chloride (0.3 mmol), the reaction mixture was stirred at rt for 7 h. The bufalin (20 mg, 0.052 mmol), DMAP (0.4 mmol) and DCE (dry, 4 mL) were added. The suspension was stirred at 100 ^{o}C for 2 h and then rt for 15 h. The mixture was then washed with 1 N HCl (aq, x 3) and brine, purified by a silica gel flash chromatography (DCM/MeOH = 30:1 to 10:1) to afford target compound (18.0 mg, 57% yield) as a white solid.

^{1}H NMR (400 MHz, $CDCl_3$) δ 7.83 (dd, *J* = 9.6, 4.0 Hz, 1H), 7.23 (s, 1H), 6.26 (d, *J* = 9.6 Hz, 1H), 5.40 (s, 1H), 5.12 (s, 1H), 5.09 (s, 1H), 4.52-4.49 (m, 1H), 4.33-4.30 (m, 1H), 3.16-3.15 (m, 1H), 2.92 (dd, *J* = 12.8, 4.8 Hz, 1H), 2.74 (d, *J* = 12.8 Hz, 1H), 2.45-2.44 (m, 1H), 2.33 (t, *J* = 7.6 Hz, 2H), 2.20-2.17 (m, 1H), 2.09-1.94 (m, 1H), 1.96-1.81 (m, 2H), 1.80-1.35 (m, 24H), 1.21 (s, 3H), 0.70 (s, 3H); ^{13}C NMR (100 MHz, $CDCl_3$) δ 173.0, 163.5, 162.3, 148.6, 146.7, 122.6, 115.3, 85.3, 70.3, 62.1, 60.2, 55.3, 51.3, 48.4, 42.4, 40.9, 40.5, 36.9, 35.9, 35.2, 34.3, 32.8, 30.6, 30.5, 29.7, 29.5, 28.7, 26.4, 25.5, 25.1, 23.8, 21.4, 21.3, 16.5; ESI-HRMS: calcd. for $C_{34}H_{49}N_2O_6S^+$ $(M+H)^+$ 613.3306, found 613.3307.

Fig.S5 CDK9 is a direct target of acetyl-bufalin

A. Viability of PC-9 cells exposed to biotin-labeled bufalin as determined by MTT assay. **B**. Representative image of an experimental microarray (blue = negative control, red = positive control, yellow= positive spot). **C.** The expression of proteins involved in the CDK9/STAT3 pathway in H460 and PC-9 cells was examined by Western blotting after acetyl-bufalin (Ac-bu) and SNS-032 (SNS) treatment, and the relative intensity was calculated. *p < 0.05, **p < 0.01, ***p <0.00, ****P<0.0001.

Fig.S6 Acetyl-bufalin inhibited the CDK9/STAT3 signaling pathway

A. CDK9 and STAT3 expression levels by western blotting after transduction with CDK9 siRNA in H460 cells, the relative intensity was calculated. **B.** Western blot analysis of CDK9 and STAT3 expression levels in H460 and PC-9 cells transfected with CDK9 siRNA1 and treated for 30 min with or without IL-6 (25 ng/ml). The relative intensity was calculated. *p < 0.05, **p < 0.01, ***p <0.00, ****P<0.0001.

Fig.S7 High CDK9 expression in human NSCLC tissues

A. Protein expression of CDK9 in each paired human NSCLC tissue sample (T) and adjacent normal lung tissues (N) from the same patient was detected by western blotting. **B**. Immunohistochemical analysis for P-CDK9 from human NSCLC tissue samples and normal tissue samples.

Supplementary Table 1: IC50 of the main active components of cinobufagin in different NSCLC cells.

Supplementary Table 2: The clinicopathologic characteristics of samples

Supplementary Table 1

IC_{50} of main active components of cinobufagin in different NSCLC cells

**Drug**	**IC_{50} μM**		
	**PC-9**	**A549**	**H460**
Arenobufagin	0.46	0.01274	
Bufalin	0.1409	0.08323	0.06472
Bufotalin	0.8133		
Pseudobufarenogin	> 100	4.268	
Cinobufagin	0.4144		
Cinobufotalin	0.5868	0.8186	
Desacetylcinobufagin	10.06	1.818	
Desacetylcinobufotalin	2.888	0.8867	
Gamabufotalin	0.281	0.7302	
Resibufogenin	4.64	1.129	1.061
Telocinobufagin	0.5741	0.1457	
Marinobufagin	15.00	1.547	1.301
Resibufagin	16.02		

Supplementary Table 2

The clinicopathologic characteristics of samples

Number	Age	Sex	Pathological diagnosis	TNM
1	63	Male	Adenocarcinoma	T1bN0M0
2	59	Male	Squamous cell carcinoma	T3N0M0
3	52	Female	Adenocarcinoma	T1bN0MX
4	64	Female	Adenocarcinoma	T1cN1MX
5	71	Male	Adenocarcinoma	T2aN2M0
6	67	Female	Adenocarcinoma	T1bN0MX
7	74	Female	Squamous cell carcinoma	T4NXM0
8	66	Female	Adenocarcinoma	T1bN0M0
9	79	Female	Adenocarcinoma	T1cN0M0
10	68	Male	Adenocarcinoma	T1bN0M0
11	60	Male	Adenocarcinoma	T1bN0MX
12	54	Male	Adenocarcinoma	T1bN0M0
